# Practice and impact of selection and centralization for oral cancer services: a scenario analysis

**DOI:** 10.1186/s12885-026-15617-y

**Published:** 2026-01-28

**Authors:** Hironori Sakai, Akinobu Shibata, Kazuya Miyamoto, Kiriko Matsuzawa, Hiroki Otagiri, Hiroshi Kurita

**Affiliations:** Department of Dentistry and Oral Surgery, Shinshu University School of Medicine 3-1-1 Asahi, Matsumoto, 390-8621 Japan

**Keywords:** Quality of life, Survival rate, Mouth neoplasms, Treatment outcome

## Abstract

**Background:**

Centralization of cancer treatment may potentially improve cancer outcomes, enhance quality of life, and reduce complications; however, few scientific studies have demonstrated its effectiveness. We aimed to define a cancer care delivery system with the aid of selection/ centralization scenarios to enhance outcomes for oral cancer, which is recognized as a rare malignancy in Japan. The purpose of this study was to review our scenarios and discuss their effectiveness.

**Methods:**

We confirmed and implemented the following pragmatic scenarios: (A) early-stage cancer can be treated at any hospital, (B) Stage III cases should be treated at cancer care hospitals, and (C) Stage IV and recurrent cases should be treated at the regional cancer care center. Participants included oral and maxillofacial surgeons in one prefectural cancer care hospital, eight regional cancer care hospitals (out of 11 base hospitals), and 10 other noncancer treatment hospitals. Medical records between 2017 and 2021 were examined. In addition, treatment outcomes from eight hospitals that had provided oral cancer treatment between 2000 and 2011 were examined to compare pre- and post-scenario treatment outcomes. Survival rates were analyzed via Kaplan-Meier estimation of 5-year overall survival (OS) and assessed for significant differences via the log-rank test. A *P*-value of less than 0.05 was considered to indicate a significant difference. All analyses were performed using JMP ver.13.2 (SAS Institute Inc., Cary, NC, USA).

**Results:**

Four hundred sixty-nine patients with oral malignancies were seen at participating hospitals. A total of 158 (33.7%) patients were subsequently referred to other hospitals for cancer treatment. A comparison of survival rates stratified by hospital category showed no significant differences between hospital categories for early-stage cancers. In contrast, there were significant differences between groups in stages III and IV. A pre- and post-scenario comparison revealed that survival rates had improved, especially for those with advanced cancer (stage IVB).

**Conclusion:**

The results of this study suggest that the scenarios we developed are useful and may improve the outcomes of oral cancer.

## Background

Centralization of cancer treatment has been thought to be effective in improving cancer treatment outcomes, enhancing quality of life, and reducing complications [1–5]. However, there are several challenges in the implementation of centralized cancer services, such as increased patient burden, understanding and cooperation of physicians, and shortcomings of the healthcare system [6–10]. These challenges warrant further development to foster better clinical decision-making and outcomes.

In Japan, oral cancer is considered one of rare cancers with an incidence rate that is generally less than 6 cases per 100,000 population [11]. Due to the rarity of the disease, particularly in rural areas, healthcare providers face difficulties in gaining clinical experience. Problems associated with rare cancers include (1) lack of experience, information, education, and training, (2) small number of basic and clinical research, (3) scarcity of established and evidence-based standard of treatment/care, and (4) fewer opportunities for developing new treatments [12]. To overcome these issues, a medical care and training system should be established in order to improve diagnosis and treatment, centralize case selection, develop human resources, and eliminate regional disparity.

Nagano prefecture, where Shinshu University Hospital is located, is a prefecture with a total area of 14,600 square kilometers and a population of approximately 2 million. The region is located approximately 200 km away from urban centers of Japan (Tokyo and Nagoya) where the population and medical facilities are concentrated. Known for its wide expanses of rural and mountainous terrain, Nagano prefecture is the fourth largest in land area among Japan’s 47 prefectures. Despite its size, it ranks only 38th in population density. The region is divided into ten secondary medical care areas, with hospitals serving as cancer treatment centers within each zone.

Medical facilities in Nagano prefecture that are capable of treating cancer were categorized into the following categories: one prefectural cancer care hospital, ten regional cancer care hospitals, and cooperative non-cancer care hospitals. Prefectural cancer care hospitals were defined as tertiary hospitals capable of comprehensive cancer care, including specialized diagnostics, complex therapies, and research. Regional cancer care hospitals were defined as secondary hospitals capable of common diagnostics and cancer therapies. Cooperative non-cancer care hospitals were defined as those capable of collaborating with cancer care hospitals but are unable to provide specialized cancer treatment. Eight of these medical institutions were capable of providing radiotherapy.

As of June 2024, Nagano Prefecture is served by 30 medical oncologists certified by the Japanese Society of Clinical Oncology. Furthermore, 17 oral surgeons have undergone general oncology training, with three holding certifications as oral cancer specialists from the Japanese Society for Oral Oncology. All three specialists are currently practicing within Nagano Prefecture. The status quo of oral cancer treatment/care is insufficient to meet the existing demands of the area, and a system needs to be established to make effective allocation and use of these limited medical resources. In addition, because there are only approximately 100 oral cancer cases per year in Nagano prefecture and medical facilities are scattered over a large area, the number of cases treated per facility as well as the opportunities for training and experience in oral cancer treatment/care are limited. The centralization of oral cancer patients is necessary to provide advanced medical care and to develop human resources, which results in improvements in the quality and efficacy of cancer treatment/care.

Japan’s healthcare system is distinguished by universal coverage and a free access model, allowing individuals to seek medical attention promptly when symptoms arise. This approach, however, may lead to either patient concentration at certain hospitals or broad dispersion across facilities. Ideally, hospitals experiencing high volumes of patients would also possess ample medical resources; in practice, this alignment does not always occur. The provision of clear and relevant information regarding specialized treatment options at various institutions is essential, as insufficient guidance can result in patients presenting at inappropriate facilities. To ensure effective care that aligns with disease progression, a more nuanced division of responsibilities among regional medical institutions and robust inter-facility collaboration are necessary. Nevertheless, unrestricted patient access often results in individuals choosing hospitals based on convenience, which can present challenges for coordinated care between institutions.

We have been working to improve the treatment/care of oral cancer patients in rural areas of Nagano prefecture by implementing selection/centralization scenarios to efficiently stratify patients. In this study, we aimed to define a cancer care delivery system with the aid of selection/centralization scenarios to enhance outcomes for oral cancer, which is recognized as a rare malignancy in Japan. In this paper, we present and discuss the implementation status and effectiveness of the scenarios.

## Materials and methods

### Selection/centralization scenarios (Fig. 1)

**Fig. 1:**
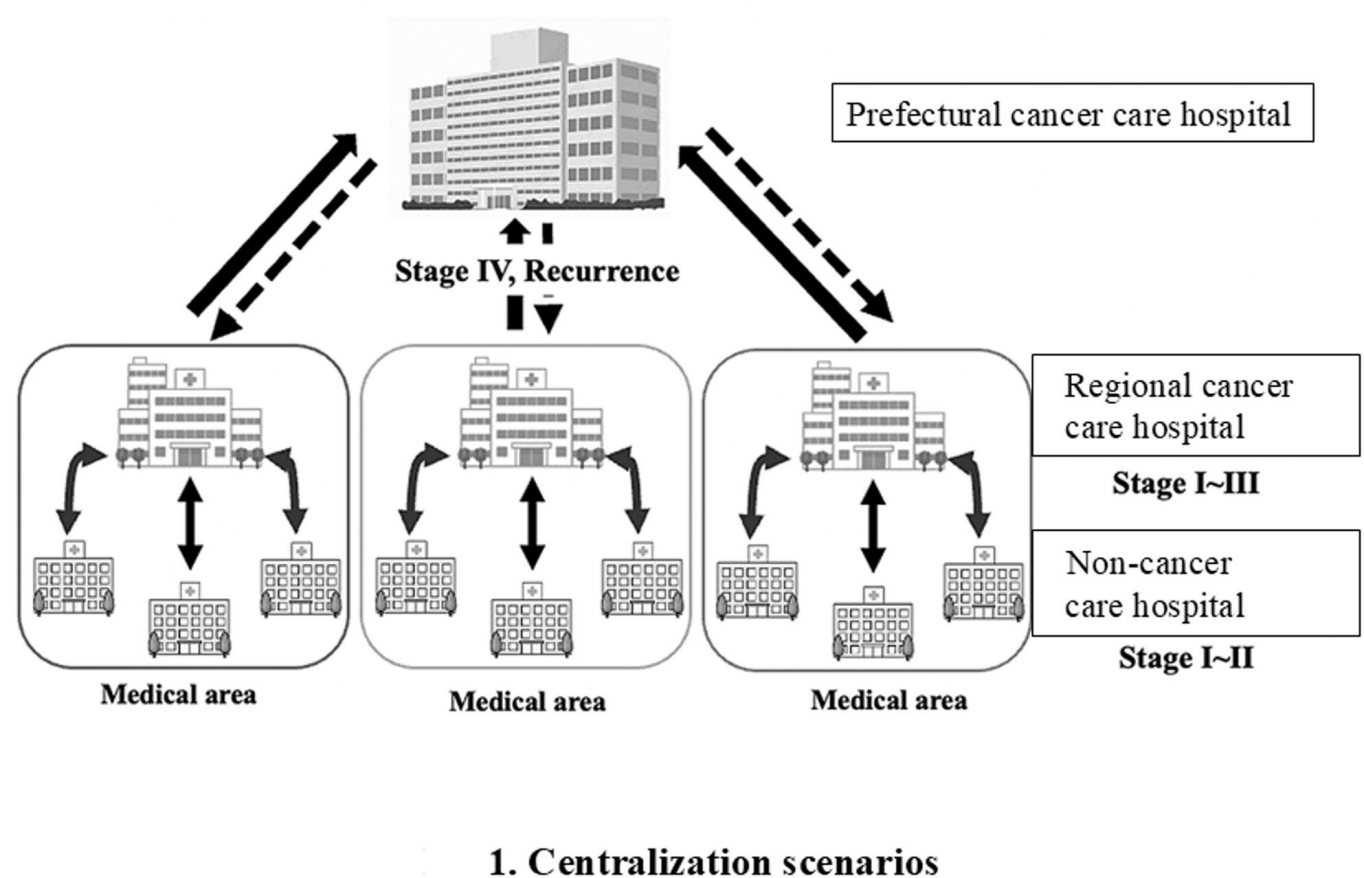
Selection/centralization scenarios

In 2025, we confirmed and implemented the following pragmatic scenarios based on a review of past treatment outcomes and problems in oral cancer treatment and available medical resources for oral cancer treatment in the prefecture.

1. Early-stage cancer (carcinoma in situ [CIS]; stages I and II) can be treated at any hospital.
2. Stage III cases should be treated at regional or prefectural cancer care hospitals.
3. Stage IV and recurrent cases should be treated at the prefectural cancer care hospital.
4. The above decisions are based on the patient’s consent and are made based on the tolerability of active treatment, travel distance, family/social support, and other factors.

### Survey on the implementation and outcome of the selection/centralization scenarios

Participants of the selection/centralization scenarios included oral and maxillofacial surgeons in one prefectural cancer care hospital (core hospital), eight regional cancer care hospitals (out of 11 base hospitals), and 10 other noncancer treatment hospitals. The Ministry of Health, Labour and Welfare of Japan defines noncancer treatment hospitals as facilities that provide routine and maintenance medical care, base hospitals as multidisciplinary acute medical care centers capable of detailed examination/confirmative diagnosis, and core hospitals as those capable of particularly advanced medical care such as cancer genome medicine.

The selection/centralization scenarios were implemented from 2017 to 2021. Medical records were reviewed to evaluate patient visit routes, treatment coordination, specific treatment details, and outcomes, including overall survival time. Hospital visits, referral pathways, treatment delivery, and results were subsequently reviewed. Additionally, pre-scenario outcome data from eight hospitals providing oral cancer care between 2000 and 2011 were collected to enable comparison with outcomes following selection and centralization.

Survival rates were analyzed via Kaplan-Meier estimation of 5-year overall survival (OS) and assessed for significant differences via the log-rank test. Survival time was calculated as the number of days between the first visit to the treatment hospital and the date of last confirmation. In general, efforts were made to start treatment within three weeks of the first visit to the hospital where the patient was first seen. A *P*-value of less than 0.05 was considered to indicate a significant difference. All analyses were performed using JMP ver.13.2 (SAS Institute Inc., Cary, NC, USA).

The study protocol was approved by the Ethics Committee of Shinshu University School of Medicine (Approval number #5251). This study was performed in accordance with the Declaration of Helsinki (2013 Fortaleza revision) and Ethical Guidelines for Medical Research for Humans.

## Results

**Fig. 2:**
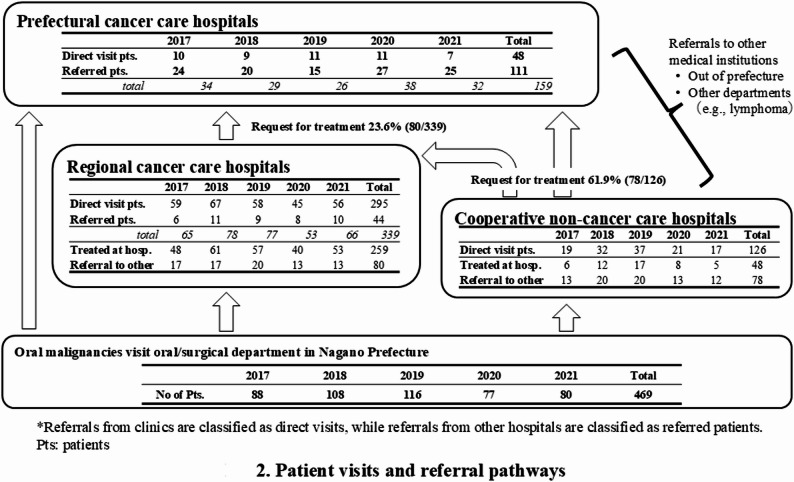
Patient visits and referral pathways

During the period between 2017 and 2021, 469 patients with oral malignancies were seen at the participating hospitals. The histological type was oral squamous cell carcinoma (SCC) in 401 cases, followed by salivary gland malignancy in 23, malignant lymphoma in 10, melanoma in 4, and sarcoma in 3. The data concerning sex, age, and primary sites are shown in Table 1. The patient visits and referral pathways are summarized in Fig. 2. Referral from the clinic was classified as a direct visit, whereas referral from a hospital was classified as a referred patient. There were 126 patients with oral malignancies in noncancer core hospitals, 48 (38.1%) of whom were treated on-site and 78 (61.9%) of whom were referred to the prefectural or the regional cancer care hospitals. A total of 339 patients with oral malignancies visited the regional cancer care hospitals, including 295 direct patients and 44 (13.0%) referred patients. Of these, 259 (76.4%) were treated at the hospitals and the remaining 80 (23.6%) were referred to the prefectural cancer care hospital for intensive treatment. The prefectural cancer care hospital treated 159 cases, of which 111 (69.8%) were referred from other hospitals. Overall, a total of 158 cases (33.7%) were referred to other hospitals for cancer treatment.

**Table 1:** Description of patients seen during post-scenario period (2017–2021)

Characteristic	Value
Sex (male: female)	241:228
Mean age	73.1 ± 14.3 years old (20–102 years old)
Primary site	
Mobile tongue	203
Lower jaw	85
Upper jaw	55
Labial/cheek mucosa	47
Oral floor	32
Soft palate/Oropharyngeal	19
Hard palate	16
Neck	7
Other	5
Cancer Stage (only SCC)	
Early stage (CIS, I, II)	233
Advance stage (III, IV)	168
Histological type	
SCC	401
Salivary gland Ca.	23
Malignant lymphoma	10
Melanoma	4
Sarcoma	3
Other (including unknown)	28

**Fig. 3:**
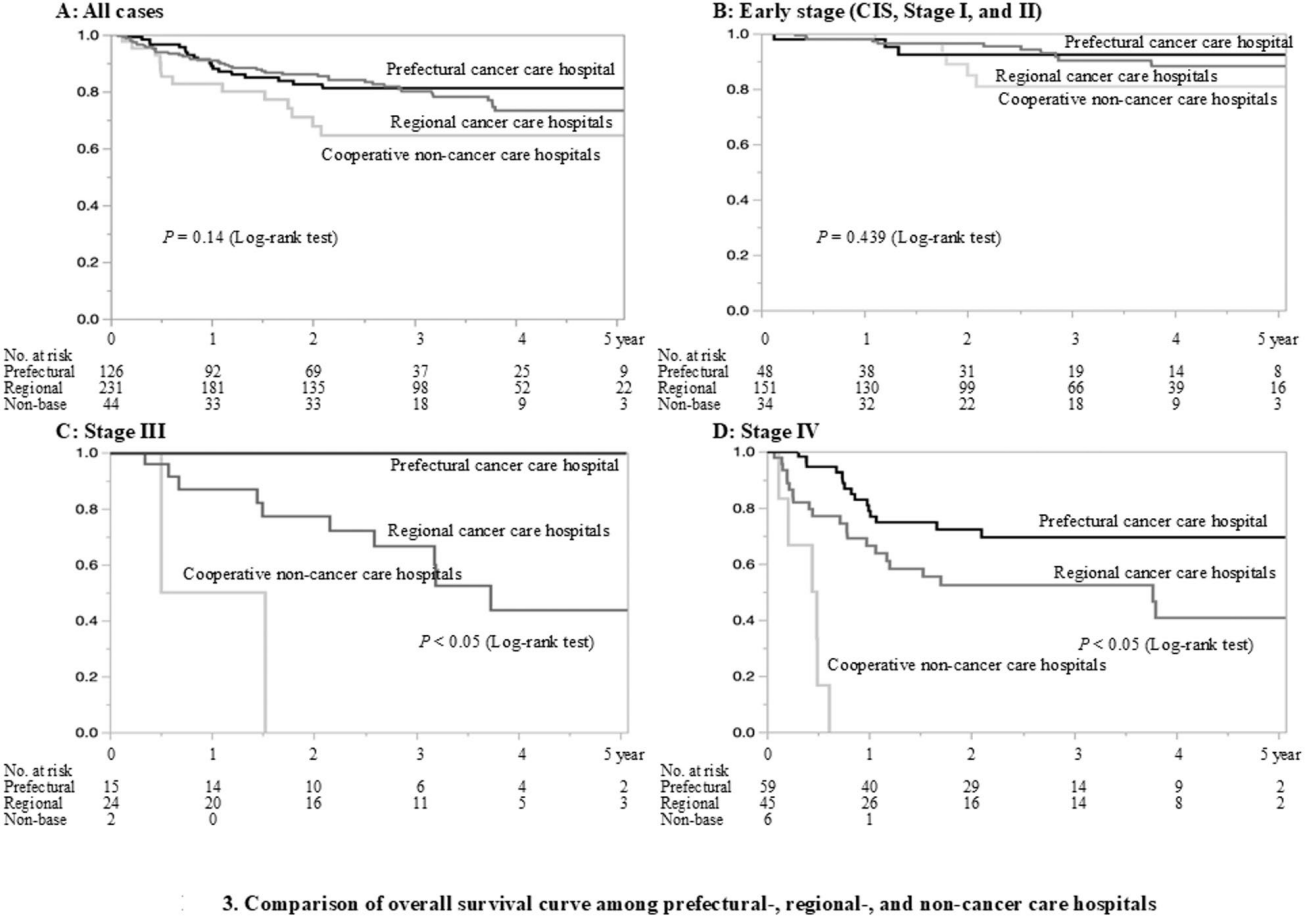
Comparison of survival rates, stratified by hospital category **A**; All cases **B**: Early Stage (C IC, Stage Ⅰ and Ⅱ) **C**: Stage Ⅲ **D**: Stage Ⅳ

The following is a review of treatment outcomes for patients with squamous cell carcinoma (401 cases) between 2017 and 2021. The characteristics and staging classification (Union for International Cancer Control [UICC] 7th ) [13] of patients treated in each group of hospitals are shown in Table 2. In the cooperative non-cancer care hospitals, 44 cases were treated, of which 77.3% (34 cases) were early-stage cancers (CIS, stages I and II). In regional cancer care hospitals, 231 cases were treated: 151 cases (65.4%) with early-stage cancer, 24 (10.4%) with stage III, and 45 (19.5%) with stage IV. At the prefectural cancer care hospital, 126 cases were treated: 48 (38.1%) with early-stage cancer, 15 (11.9%) with stage III, and 59 (46.8%) with stage IV. The 3-year and 5-year cumulative OS rates for all patients were 78.3% and 74.1%, respectively. The 5-year cumulative OS was 88.0% for early-stage cancer, 56.0% for stage III (41 cases), and 51.8% for stage IV (110 cases). A comparison of survival rates, stratified by hospital category, is summarized in Fig. 3. For early-stage cancer, there was no significant difference among the hospital categories (5-year cumulative OS: 92.4% for the prefecture, 88.3% for the region, and 80.9% for cooperative non-cancer care hospitals; log-rank test, *P* = 0.43). On the other hand, in stages III and IV, there was a significant difference among the groups (5-year cumulative OS in stage III: 100% for the prefecture, 43.7% for the region, and 0% for the cooperative non-cancer care hospitals; log-rank test, *P* < 0.05; stage IV: 69.5%, 40.7%, and 0%, respectively; log-rank test, *P* < 0.05).

**Table 2:** Comparison of oral squamous cell carcinoma characteristics between post-scenario (2017–2021) and pre-scenario (2000–2011) periods

	2017–2021	2000–2011	
Number of cases	401	461	
Sex (Male: Female)	214:187	260:201	*P* = 0.37 ^†^
Mean age	72.6 +- 14.1	67.9 +- 14.5	*P* < 0.05 ^*^
Primary site:			*P* = 0.18 ^†^
Mobile tongue	199 (49.6%)	197 (42.7%)	
Lower gum	65 (16.2%)	93 (20.2%)	
Upper gum	45 (11.2%)	71 (15.4%)	
Lip and buccal mucosa	38 (9.5%)	38 (8.2%)	
Oral floor	27 (6.7%)	36 (7.8%)	
Oropharyngeal mucosa	13 (3.2%)	17 (3.7%)	
Lip	3 (0.7%)	4 (0.9%)	
Hard palate	6 (1.5%)	2 (0.4%)	
Others	5 (1.2%)	3 (0.7%)	

^*^ Student t-test, ^†^ Pearson chi-square test

**Fig. 4:**
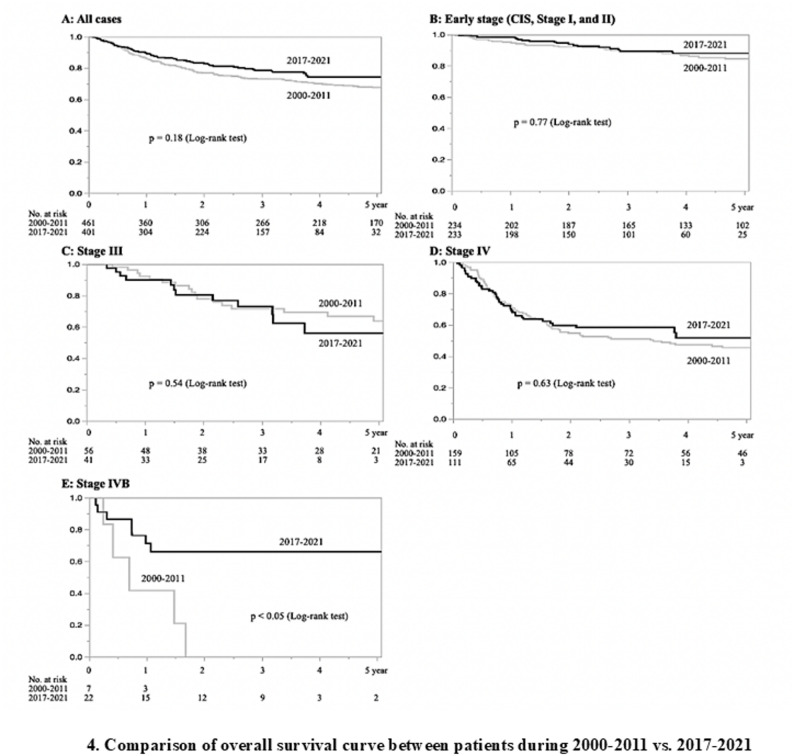
Comparison of treatment outcomes before and after the scenarios **A**; All cases **B**: Early Stage (C IC, Stage Ⅰ and Ⅱ) **C**: Stage Ⅲ **D**: Stage Ⅳ **E**: Stage ⅣB

A comparison of the treatment outcomes of patients with oral squamous cell carcinoma before (2000–2011) and after (2017–2021) the scenarios are presented below. The characteristics of the patients in both periods are shown in Table 2. There was a significant difference in the mean age between the two periods, indicating an increase in the number of older patients in 2017–2021. No significant differences in sex or primary tumor distribution were found. A comparison of the clinical stages of oral cancer before and after the selection/centralization scenarios is shown in Table 3. There was an increase in early-stage cancer cases after 2017 (50.8% vs. 58.1%) and a decrease in advanced stage cancer cases (46.6% vs. 37.9%) compared with those before 2011. When compared between hospital categories, there was a decrease in early-stage cancers (42.8% vs. 38.1%) and an increase in advanced cancers (52.3% vs. 58.7%) in the prefectural cancer care hospital. On the other hand, in the regional cancer care hospitals, an increase in early-stage cancers (60.1% vs. 65.4%) and a decrease in stage IV cancers (28.7% vs. 19.5%) were observed. In the cooperative non-cancer care hospitals, an increase in early-stage cancers (58.7% vs. 77.3%) and a decrease in advanced-stage cancers (41.3% vs. 20.4%) were observed. Figure 4 summarizes the comparison of treatment outcomes before and after the scenarios. Overall, there was an improvement in 5-year OS after centralization, but the difference was not significant (67.6% vs. 74.1%; log-rank test, *P* = 0.18). For early-stage cancer, there was no difference in 5-year OS between before and after the scenario 84.5% vs. 88.0%), and for stage III, the survival rate was lower after 2017 than before 2011 (63.7% vs. 56.0%). An improvement in 5-year OS was obtained for stage IV patients, although it did not reach statistical significance (45.5% vs. 51.8%; log-rank test, *P* = 0.64). When comparing the OS of stage IVB, which is considered to have a poor outcome, a significant improvement in OS was achieved after the implementation of selection/centralization scenarios (0% vs. 66.0%; log-rank test, *P* < 0.05).

**Table 3:** Staging classification (UICC 7th) of patients treated in each group of hospitals between 2000–2011 and 2017–2021

		*n*	Early stage^*^	Stage III	Stage IV	Recurrence /others
2017–2021						
Prefectural cancer care hospital (1)		126	38.1%	11.9%	46.8%	*3.2%*
Regional cancer care hospitals (8)		231	65.4%	10.4%	19.5%	*4.7%*
Cooperative non-cancer care hospitals (10)		44	77.3%	4.5%	15.9%	*2.3%*
All		401	58.1%	10.2%	27.7%	*4.0%*
2000–2011						
Prefectural cancer treatment hospital (1)		243	42.8%	10.3%	42.0%	*4.9%*
Regional cancer treatment hospitals (4)		143	60.1%	11.2%	28.7%	*0%*
Cooperative non-cancer care hospitals (3)		75	58.7%	20.0%	21.3%	*0%*
All		461	50.8%	12.1%	34.5%	*2.6%*

^*^ Carcinoma in situ, Stage I and II

## Discussion

We have attempted to clarify cancer medical care delivery system (the selection/centralization “scenarios”) to improve outcomes for oral cancer, which is one of the rare cancers in Japan and a disease with many treatment challenges especially in rural areas. The purpose of this study was to review our scenarios and discuss their effectiveness. We consider this report to be the first to assess the impact of selection/centralization of oral cancer care, which is a strength of this study.

One of the scenarios was the centralization of cases of advanced oral cancer to hospitals specializing in cancer treatment. The flow of patients in 2017–2021 (after the scenario was developed) revealed that, overall, 33.7% (158/469 cases) were referred to hospitals specialized in cancer treatment. A total of 61.9% of cases in the cooperative non-cancer care hospitals and 23.6% of those in the regional cancer care hospitals were referred to higher-level cancer care hospitals. In the comparison of the clinical stages of treated cases in each hospital category between 2017 and 2021 and 2000–2011, a decrease in stage III and IV cases in cooperative non-cancer care hospitals and a decrease in stage IV cases in regional cancer care hospitals were observed. On the other hand, the proportion of early-stage cancers among treated cases has increased in the cooperative non-cancer and regional cancer care hospitals since 2017 compared with before 2011, suggesting that our designed patient selection and centralization scenarios have been progressing.

While the centralization of cancer treatment is expected to have positive impacts, it also poses many challenges. Centralization increases patient travel and financial burdens. There are also issues on the patient side, including patient age, place of residence, family structure, general condition (morbidity), and willingness to receive treatment.

In the 2017 and later data, stage IV cases were still treated in the cooperative non-cancer care hospitals and regional cancer care hospitals (stage IV accounted for 15.9% in the cooperative non-cancer care hospitals and 19.5% in the regional cancer care hospitals). These patients were those who were unwilling or unable to travel because of their age, family support, and general health (comorbidity). The number of elderly cancer patients is increasing. As the scenario of centralization in oral cancer treatment advances, it is necessary to consider measures for the management of elderly oral cancer patients.

After the selection/centralization scenarios, the treatment outcome was favorable, with a 5-year cumulative survival rate of 74.1%. The assessment of treatment outcomes revealed that there was no difference in treatment outcomes for early-stage cancers among the prefectural, regional, or non-cancer care hospitals, whereas significant differences were observed for stage III and IV cancers. These results suggest that our planned scenario demonstrates the need for centralization of care in advanced cases. As mentioned earlier, these differences may have arisen because non-cancer and regional cancer care hospitals often treat older patients and patients in poor general condition. However, it is reasonable to assume that hospitals specializing in cancer care have facilities and specialists to improve outcomes. The treatment of oral cancer is multidisciplinary and requires advanced treatment facilities and specialists. There are no reports examining the effect of centralization on oral cancer, but beneficial effects have been reported in other cancers [1–5]. The treatment of oral cancer is becoming increasingly complex. One effective way to improve the outcomes and quality of oral cancer services would be to promote centralization.

A comparison of treatment outcomes before and after centralization scenarios revealed a 6.5% increase in 5-year OS after scenario development, although this increase was not statistically significant. The results revealed no difference in outcomes of early-stage cancers, but improved outcomes of advanced cancers (stage IV). In particular, stage IVB cases were consolidated in prefectural cancer care hospitals, and a significant improvement in the 5-year OS was evident, which we believe is a result of the effective functioning of the scenarios we developed. It is possible that improved oral cancer outcomes may be achieved over time. In oral cancer treatment, the introduction of molecularly targeted drugs (cetuximab) and immune checkpoint inhibitors began in 2012 in Japan, and this change may be a factor in improved outcomes. Both drugs are recommended for use in specialist oncology hospitals, and centralization of advanced cases may also have some role.

## Conclusions

The results of this study suggested that the scenario we have developed is working and that it may also improve treatment outcomes. Concentrating rare cancers could also lead to advances in clinical research. In rare diseases such as oral cancer, case selection and centralization may be important strategies.

The strength of this study is that, for the first time in oral cancer, the effects of case selection and centralization were examined. However, the study does not yet demonstrate a significant overall improvement in survival. Many issues need to be addressed in the centralization of oral cancer services. Specifically, concerns regarding the centralization of cancer treatment include increased patient burden, the need for physician understanding and cooperation, and deficiencies in the healthcare system. It is necessary to continue to advance case selection and centralization and to study its effects while seeking ways to resolve these issues.

### Limitations

This study did not investigate HPV infection.

## Data Availability

The datasets generated during and/or analyzed during the current study are available from the corresponding author upon reasonable request.

## Abbreviations

CIS: carcinoma in situ
OS: overall survival
SCC: squamous cell carcinoma
UICC: Union for International Cancer Control
